# Supplementary material for: A fault-intrusion-tolerant system and deadline-aware algorithm for scheduling scientific workflow in the cloud
Source: PeerJ Comput Sci. 2021 Nov 2;7:e747. doi: 10.7717/peerj-cs.747 (PMC8576554; doi:10.7717/peerj-cs.747)
Supplement: Supplemental Information 6 [file peerj-cs-07-747-s006.zip › Myproject1.1/docs/org/cloudbus/cloudsim/power/PowerHost.html]

PowerHost


JavaScript is disabled on your browser.


- Overview
- Package
- Class
- Use
- Tree
- Deprecated
- Index
- Help

- Prev Class
- Next Class

- Frames
- No Frames

- All Classes

- Summary:
- Nested |
- Field |
- Constr |
- Method

- Detail:
- Field |
- Constr |
- Method


org.cloudbus.cloudsim.power

## Class PowerHost

- java.lang.Object
- - org.cloudbus.cloudsim.Host
  - - org.cloudbus.cloudsim.HostDynamicWorkload
    - - org.cloudbus.cloudsim.power.PowerHost

- Direct Known Subclasses:
  :   PowerHostUtilizationHistory

  ---

    

  ```
  public class PowerHost
  extends HostDynamicWorkload
  ```

  PowerHost class enables simulation of power-aware hosts.
  If you are using any algorithms, policies or workload included in the power package please cite
  the following paper:
  Anton Beloglazov, and Rajkumar Buyya, "Optimal Online Deterministic Algorithms and Adaptive
  Heuristics for Energy and Performance Efficient Dynamic Consolidation of Virtual Machines in
  Cloud Data Centers", Concurrency and Computation: Practice and Experience (CCPE), Volume 24,
  Issue 13, Pages: 1397-1420, John Wiley & Sons, Ltd, New York, USA, 2012

  Since:
  :   CloudSim Toolkit 2.0

- - ### Constructor Summary

    Constructors

    Constructor and Description

    `PowerHost(int id,
    RamProvisioner ramProvisioner,
    BwProvisioner bwProvisioner,
    long storage,
    java.util.List<? extends Pe> peList,
    VmScheduler vmScheduler,
    PowerModel powerModel)`

    Instantiates a new host.
  - ### Method Summary

    Methods

    | Modifier and Type | Method and Description |
    | --- | --- |
    | `double` | `getEnergyLinearInterpolation(double fromUtilization, double toUtilization, double time)` Gets the energy consumption using linear interpolation of the utilization change. |
    | `double` | `getMaxPower()` Gets the max power that can be consumed by the host. |
    | `double` | `getPower()` Gets the power. |
    | `protected double` | `getPower(double utilization)` Gets the power. |
    | `PowerModel` | `getPowerModel()` Gets the power model. |
    | `protected void` | `setPowerModel(PowerModel powerModel)` Sets the power model. |

    - ### Methods inherited from class org.cloudbus.cloudsim.HostDynamicWorkload

      `addStateHistoryEntry, getCompletedVms, getMaxUtilization, getMaxUtilizationAmongVmsPes, getPreviousUtilizationMips, getPreviousUtilizationOfCpu, getStateHistory, getUtilizationMips, getUtilizationOfBw, getUtilizationOfCpu, getUtilizationOfCpuMips, getUtilizationOfRam, setPreviousUtilizationMips, setUtilizationMips, updateVmsProcessing`
    - ### Methods inherited from class org.cloudbus.cloudsim.Host

      `addMigratingInVm, allocatePesForVm, deallocatePesForVm, getAllocatedMipsForVm, getAvailableMips, getBw, getBwProvisioner, getDatacenter, getId, getMaxAvailableMips, getNumberOfFreePes, getNumberOfPes, getPeList, getRam, getRamProvisioner, getStorage, getTotalAllocatedMipsForVm, getTotalMips, getVm, getVmList, getVmScheduler, getVmsMigratingIn, isFailed, isSuitableForVm, reallocateMigratingInVms, removeMigratingInVm, setBwProvisioner, setDatacenter, setFailed, setFailed, setId, setPeList, setPeStatus, setRamProvisioner, setStorage, setVmScheduler, vmCreate, vmDeallocate, vmDeallocateAll, vmDestroy, vmDestroyAll`
    - ### Methods inherited from class java.lang.Object

      `clone, equals, finalize, getClass, hashCode, notify, notifyAll, toString, wait, wait, wait`

- - ### Constructor Detail


    - #### PowerHost

      ```
      public PowerHost(int id,
               RamProvisioner ramProvisioner,
               BwProvisioner bwProvisioner,
               long storage,
               java.util.List<? extends Pe> peList,
               VmScheduler vmScheduler,
               PowerModel powerModel)
      ```

      Instantiates a new host.

      Parameters:
      :   `id` - the id
      :   `ramProvisioner` - the ram provisioner
      :   `bwProvisioner` - the bw provisioner
      :   `storage` - the storage
      :   `peList` - the pe list
      :   `vmScheduler` - the VM scheduler
  - ### Method Detail


    - #### getPower

      ```
      public double getPower()
      ```

      Gets the power. For this moment only consumed by all PEs.

      Returns:
      :   the power


    - #### getPower

      ```
      protected double getPower(double utilization)
      ```

      Gets the power. For this moment only consumed by all PEs.

      Parameters:
      :   `utilization` - the utilization

      Returns:
      :   the power


    - #### getMaxPower

      ```
      public double getMaxPower()
      ```

      Gets the max power that can be consumed by the host.

      Returns:
      :   the max power


    - #### getEnergyLinearInterpolation

      ```
      public double getEnergyLinearInterpolation(double fromUtilization,
                                        double toUtilization,
                                        double time)
      ```

      Gets the energy consumption using linear interpolation of the utilization change.

      Parameters:
      :   `fromUtilization` - the from utilization
      :   `toUtilization` - the to utilization
      :   `time` - the time

      Returns:
      :   the energy


    - #### setPowerModel

      ```
      protected void setPowerModel(PowerModel powerModel)
      ```

      Sets the power model.

      Parameters:
      :   `powerModel` - the new power model


    - #### getPowerModel

      ```
      public PowerModel getPowerModel()
      ```

      Gets the power model.

      Returns:
      :   the power model


- Overview
- Package
- Class
- Use
- Tree
- Deprecated
- Index
- Help

- Prev Class
- Next Class

- Frames
- No Frames

- All Classes

- Summary:
- Nested |
- Field |
- Constr |
- Method

- Detail:
- Field |
- Constr |
- Method
